# Space‐for‐time is not necessarily a substitution when monitoring the distribution of pelagic fishes in the San Francisco Bay‐Delta

**DOI:** 10.1002/ece3.8292

**Published:** 2021-11-16

**Authors:** Adam Duarte, James T. Peterson

**Affiliations:** ^1^ Pacific Northwest Research Station U.S.D.A. Forest Service Olympia Washington USA; ^2^ Department of Fisheries, Wildlife, and Conservation Sciences Oregon State University Corvallis Oregon USA; ^3^ Oregon Cooperative Fish and Wildlife Research Unit Department of Fisheries, Wildlife, and Conservation Sciences U.S. Geological Survey Oregon State University Corvallis Oregon USA

**Keywords:** Delta Smelt, incomplete capture, Longfin Smelt, occupancy model, space‐for‐time substitution, species distribution model

## Abstract

Occupancy models are often used to analyze long‐term monitoring data to better understand how and why species redistribute across dynamic landscapes while accounting for incomplete capture. However, this approach requires replicate detection/non‐detection data at a sample unit and many long‐term monitoring programs lack temporal replicate surveys. In such cases, it has been suggested that surveying subunits within a larger sample unit may be an efficient substitution (i.e., space‐for‐time substitution). Still, the efficacy of fitting occupancy models using a space‐for‐time substitution has not been fully explored and is likely context dependent. Herein, we fit occupancy models to Delta Smelt (*Hypomesus transpacificus*) and Longfin Smelt (*Spirinchus thaleichthys*) catch data collected by two different monitoring programs that use the same sampling gear in the San Francisco Bay‐Delta, USA. We demonstrate how our inferences concerning the distribution of these species changes when using a space‐for‐time substitution. Specifically, we found the probability that a sample unit was occupied was much greater when using a space‐for‐time substitution, presumably due to the change in the spatial scale of our inferences. Furthermore, we observed that as the spatial scale of our inferences increased, our ability to detect environmental effects on system dynamics was obscured, which we suspect is related to the tradeoffs associated with spatial grain and extent. Overall, our findings highlight the importance of considering how the unique characteristics of monitoring programs influences inferences, which has broad implications for how to appropriately leverage existing long‐term monitoring data to understand the distribution of species.

## Graphical abstract

We demonstrate how inferences concerning the distribution of pelagic fishes change when using a space‐for‐time substitution and occupancy models. Specifically, we found the probability that a sample unit was occupied was much greater when using a space‐for‐time substitution, presumably due to the change in the spatial scale of our inferences. Furthermore, we observed that as the spatial scale of our inferences increased, our ability to detect environmental effects on system dynamics was obscured, which we suspect is related to the tradeoffs associated with spatial grain and extent.

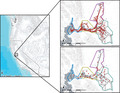

## 1 INTRODUCTION

Natural resource managers invest considerable resources to support long‐term monitoring programs to maintain a continuous flow of data on biological systems of interest. Although such data are collected for a variety of purposes, information concerning how and why species redistribute across dynamic landscapes is usually of high interest. However, it is often the case that monitoring data are compromised to some extent by the sampling or observation process. For example, it is widely recognized that our ability to monitor the distribution and abundance of fish and wildlife is complicated by incomplete capture (Bayley & Peterson, 2001; Conroy et al., 2008; Haynes et al., 2013; Pine et al., 2003; Rowe et al., 2019). That is, variation in catch data can be related to both ecological (i.e., true variation in distribution and abundance) and observational (i.e., variation in capture efficiency) processes. Failure to account for incomplete capture complicates our ability to reliably interpret patterns contained in monitoring data because it requires the likely violated assumptions that capture probabilities are constant (or only vary randomly across space and time) and that the magnitude of the biological signal in trends is greater than the noise in the monitoring data (Anderson, 2001; Thompson, 2002).

Occupancy models are probably the most widely used statistical approach to quantify the relationship between species occurrence and environmental conditions while accounting for incomplete capture (MacKenzie et al., 2017). Like all estimators, occupancy models contain a few assumptions: the occupancy state (typically species presence or absence) cannot change at a sample unit across replicate surveys; detection probabilities must be independent across sample units and replicate surveys; heterogeneity in occupancy and detection probability can be explained using covariates; and false‐positive detections do not occur. Occupancy models have proven to be an extremely flexible tool as extensions to the original parameterization continue to be rapidly developed to better suite a variety of ecological problems (reviewed in MacKenzie et al., 2017). Still, occupancy models require replicate detection/non‐detection data at a sample unit to be fitted (but see Henry et al., 2020). Such data are typically collected by surveying a focal sample unit multiple times, where replicate surveys are far enough apart in time that detection probabilities are independent and close enough in time that the occupancy state at the sample unit is static. However, long‐term monitoring programs that were initiated prior to the widespread use of occupancy models typically lack replicate surveys at a sample unit due to the fact that they often come at the cost of not being able to survey a greater number of sample units. In such cases, it has been suggested that managers can potentially treat surveys of multiple subunits within a larger sample unit as replicate surveys when fitting occupancy models (Guillera‐Arroita, 2011; Kendall & White, 2009). This space‐for‐time substitution is attractive because managers can potentially account for incomplete capture without reducing the spatial coverage of monitoring programs, and it potentially allows managers to leverage existing monitoring data that lack temporal replicate surveys at a sample unit in their sampling design. Still, fitting occupancy models to data that implement a space‐for‐time substitution has not been fully explored (Kéry & Royle, 2016).

The Sacramento‐San Joaquin Delta and San Francisco Bay (Bay‐Delta) system is the largest estuary on the Pacific Coast. Management decision making in the Bay‐Delta is complex as managers attempt to restore and conserve at‐risk fish species while trying to deliver freshwater to meet the needs of the public (Brown et al., 2009; Hanemann & Dyckman, 2009; Moyle et al., 2018). Long‐term fish monitoring programs using trawl surveys were initiated as early as the late 1950s to support decision making for various fishes in the Bay‐Delta. Over the years, additional monitoring programs that rely on trawl surveys have been initiated by different management agencies to monitor fishes for different objectives. Although fish catch data from these surveys have long been used to inform policy and management decision making, it was not until relatively recent that the effect of the observation process on inferences regarding the distribution and relative abundance of fishes in the Bay‐Delta was considered (Goertler et al., 2020; Latour 2016; Mahardja et al., 2021, 2017; Mitchell et al., 2017, 2019; Newman 2008; Peterson & Barajas, 2018; Polansky et al., 2019; Thomson et al., 2010). Overall, this collection of work has demonstrated that fish capture efficiency varies markedly across species, time, location, and monitoring program within the Bay‐Delta.

Given the above findings, it is clear that the data collected by fish monitoring programs in the Bay‐Delta need to be corrected for incomplete capture to properly inform decision making. Like many monitoring programs across the world (Budy et al., 2015; Duarte et al., 2021; Kéry et al., 2010; Sadoti et al., 2013; Van Strien et al., 2013; Whitlock et al., 2020; and many more), occupancy models are increasingly being used to accomplish this for fishes in the Bay‐Delta despite sampling designs not explicitly considering occupancy models when many of these monitoring programs were established (Goertler et al., 2020; Mahardja et al., 2021, 2017; Peterson & Barajas, 2018). However, one of the long‐term monitoring programs used to monitor the distribution and relative abundance of Delta Smelt (*Hypomesus transpacificus*) in the Bay‐Delta, the Spring Kodiak Trawl (SKT), lacks temporal replicate surveys (Newman et al., 2017). In 2016, the Enhanced Delta Smelt Monitoring Program (EDSM) was initiated to provide accurate and higher‐resolution spatial and temporal data on the distribution and relative abundance of Delta Smelt in the Bay‐Delta (Newman et al., 2017). It includes temporal replicate surveys at sample units using the same sampling gear as SKT. Our objective was to use these real‐world fish monitoring data that deploy the same sampling gear to demonstrate some of the tradeoffs associated with using spatial replicates and occupancy models to monitor variation in the distribution of species. To accomplish this, we fit occupancy models to SKT and EDSM data using both temporal and spatial replicate surveys to estimate factors related to the distribution of the two native pelagic fish species in the Bay‐Delta, Delta Smelt and Longfin Smelt (*Spirinchus thaleichthys*), which are both species of concern and often the focus of the different monitoring programs in the Bay‐Delta. We then compared the estimates among the different approaches and datasets.

## 2 METHODS

### 2.1 Study area

**FIGURE 1:**
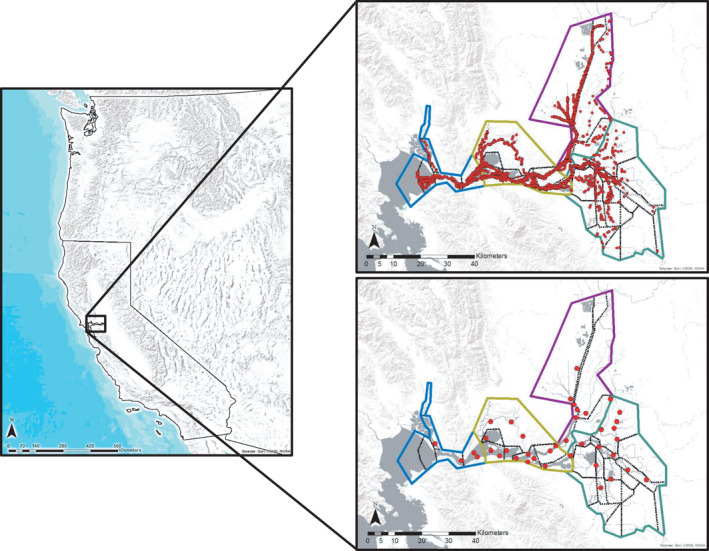
The location of the San Francisco Bay‐Delta (left) with tow locations for the Enhanced Delta Smelt Monitoring Program (top right) and Spring Kodiak Trawl (bottom right). Regions and subregions are shown using solid‐color and dashed‐black lines, respectively

The Bay‐Delta system encompasses the area that is bounded by the confluence of the Sacramento and San Joaquin Rivers to the south and east, the San Pablo Bay to the West, and Suisun Bay to the north in California, USA (Figure 1). This region supports more than 500 fish, wildlife and plant species, including several threatened and endangered species. Although the Bay‐Delta was once comprised of a series of marsh–wetland complexes in the eastern portion at the confluence of the Sacramento and San Joaquin Rivers, levee construction along stream channels and the construction of islands began in the mid‐19th century to, in part, control seasonal flooding and to protect water exports from saltwater intrusion to better meet the increasing demands for freshwater across the State of California (Galloway et al., 1999). Over the years, system‐wide changes to the environment have been documented, including a rise in contaminants (Fong et al., 2016), invasion by exotic species (Linville et al., 2002; Underwood et al., 2006), an increase in water clarity, a shift in the distance from the Golden Gate Bridge to the point where the salinity on the bottom is about 2 ppt (locally referred to as X2 and often used as a measure of water quality), and an increase in specific conductance and temperature (Peterson & Barajas, 2018).

### 2.2 Species background

Delta Smelt are endemic to the Bay‐Delta and listed as threatened by the U.S. Fish and Wildlife Service (U.S. Fish and Wildlife Service, 1993) and endangered by the State of California (California Department of Fish and Game, 2008). The species is believed to be pelagic, using primarily open water habitats (Sommer et al., 2007). Delta Smelt are described as small (i.e., individuals typically only reach about 60–70 mm in length), translucent, semi‐anadromous fish. Evidence suggests that in the wild most Delta Smelt complete an entire life cycle in just 1 year (Moyle et al., 1992). Dominant life stages available for capture by trawl surveys vary by season, with adults being most available for capture from January through March, juveniles being most available for capture from June through August, and subadults being most available for capture from September through December (Moyle et al., 2016).

Longfin Smelt are also a native pelagic fish in the Bay‐Delta, but they can be found along the Pacific coast from California to Alaska. Although the U.S. Fish and Wildlife Service found that the Bay‐Delta Distinct Population Segment of Longfin Smelt warrants consideration for protection under the U.S. Endangered Species Act, no formal federal listing decision has been made (U.S. Fish and Wildlife Service, 2012). Still, Longfin Smelt are listed as threatened by the State of California (California Department of Fish and Game, 2009). Longfin Smelt can be larger than Delta Smelt, reaching approximately 90–110 mm in length, and they are a facultative anadromous species (Rosenfield & Baxter, 2007). Longfin Smelt spawn at 2 years of age and are semelparous. Similar to Delta Smelt, dominant life stages available for capture varies by season, with adults and subadults being most available for capture from November through May and juveniles being most available for capture from June through October (Merz et al., 2013).

### 2.3 Data collection

The EDSM was initiated in 2016 by the U.S. Fish and Wildlife Service as part of their Delta Juvenile Fish Monitoring Program (U.S. Fish and Wildlife Service. Accessed August 27, 2019, https://portal.edirepository.org/nis/mapbrowse?packageid=edi.415.1). The objective of EDSM is to provide accurate and high‐resolution data on the distribution and relative abundance of Delta Smelt using two different gears: the Kodiak trawl and 20 mm survey. We did not analyze EDSM data collected by the 20 mm survey for this study because it uses a different sampling gear than the SKT. Surveys using the Kodiak trawl began in late 2016 by dividing the Bay‐Delta into strata and using a generalized random‐tessellation stratified design (Stevens & Olsen, 2004) with equal probability sampling to select sampling locations. The program began with four strata, but these strata were refined into eight strata in July 2017 and then again into 10 strata in December 2017. After inspection of the data, however, we noticed that nonrandom patterns in location selection seemed to occur prior to July 2017, which may be related to targeted surveys for other purposes (Shawn Acuña, Metropolitan Water District of Southern California, personal communication). Tows for the Kodiak trawl occurred in every month except April and May and used a net that has a 13.9 m^2^ mouth opening and a mesh size that ranges from 5.08 cm at the mouth to 0.64 cm at the end. The EDSM implements replicate tows to characterize the presence, absence, and relative abundance of fish at each sampling location. Because of how replicate tows are implemented, a sample unit is the general area around each randomly generated sampling location. Importantly, prior to sampling, the EDSM protocol sets the maximum number of replicate tows that can be taken at a sample location depending on presumed Delta Smelt density and time period of the survey. However, EDSM uses a modified removal design where replicate samples are terminated or tow duration reduced based on the number of Delta Smelt that are captured in the first two tows at a sample location to minimize detrimental effects of sampling on Delta Smelt in the sample unit. When and how often this occurred is not readily available in the metadata. Each tow was classified based on the amount of debris in the net and the time the net was in the water. We restricted our analyses to data collected from July 2017 through March 2019 and condition 1 tows (i.e., tows that were “good/normal,” with no blockage of the net and ≥3 min long; Figure 1). This dataset had 2369 sample units with an average of 4.43 replicate tows (range: 1–9) at each sample unit. Before and after each tow, field crews collected environmental data. Specific conductivity (μs/cm^3^), water turbidity (NTU), water temperature (°C), and dissolve oxygen (mg/L) at the surface were measured with calibrated meters. Secchi depth (m) and bottom depth (m) at the beginning of the tow also were measured and recorded. At the end of each tow, field crews recorded the tow duration (min), the tow number at the site that day, the volume of water sampled (m^3^), the time of sampling, the start location for the tow, tow direction (upstream, downstream, or neither), and the number of fish caught at different sizes for each species. Although tide data were recorded, these data were lacking for 16.36% of the tows. Therefore, we downloaded tide height data from the National Oceanic and Atmospheric Administration (NOAA) station at Port Chicago, CA (NOAA. Accessed May 11, 2020, https://tidesandcurrents.noaa.gov/stationhome.html?id=9415144), and classified tides as flood, ebb, and slack based on changes in tide height. Importantly, we used a threshold of 0.04 m per 15 min change to indicate a change in the tide direction to avoid high turnover in tide classifications due to minor changes in tide height. We used these tide data for all EDSM tows to maintain consistency in the source of the data. We examined the environmental data for outliers that may have been caused by equipment malfunction and treated these values as missing data per the suggestion of the EDSM data curator (Catherine Johnston, U.S. Fish and Wildlife Service, personal communication). To compare approaches (i.e., spatial vs. temporal replicates using occupancy models) with these data, our primary analysis of EDSM data used temporal replicate surveys on the same day at a sampling location as the replicate surveys in a sample unit, and our secondary analysis of EDSM data included both spatial and temporal replicate surveys within a subregion on the same day as the replicate surveys in the sample unit (hereafter referred to as EDSM with spatial replicates; Figure 1). Importantly, we opted to include both spatial and temporal replicate surveys within a subregion for the EDSM with spatial replicates analysis to make more direct comparisons with our primary EDSM analysis by having the exact same data included. This resulted in a dataset with 1450 sample units with an average of 7.24 replicate tows (range: 1–24) at each sample unit.

The SKT was initiated in 2002 by the California Department of Fish and Wildlife to monitor the distribution and relative abundance of spawning Delta Smelt (California Department of Fish and Wildlife. Accessed April 15, 2020, http://www.dfg.ca.gov/delta/projects.asp?ProjectID=SKT). SKT samples fish once a month from January through May at 40 fixed stations (Figure 1). Although supplemental tows have occurred opportunistically over the years, we restricted our analyses to data collected by the formal tows at the fixed sites of the SKT from January 2002 through May 2019. Notably, all sample data were included because tow condition information similar to EDSM are not available for these data. Samples were collected using a 10‐min surface tow while deploying almost the identical net used in the EDSM Kodiak trawls. Before and after each tow, field crews collected environmental data. Specific conductivity (μs/cm^3^), water turbidity (NTU), and water temperature (°C) at the surface were measured with calibrated meters. Secchi depth (m) and bottom depth (m) also were measured and recorded. Tides were recorded as: high (slack), ebb, low (slack), and flood. At the end of each tow, field crews recorded the tow distance (m) and duration (min), the volume of water sampled (m^3^), the time of sampling, and the number of fish caught for each species. Again, we examined the environmental data for outliers that may have been caused by equipment malfunction and treated these values as missing data. We also treated tow volume measurements as missing data when the SKT metadata noted that there was a malfunction with the flow meter used to calculate tow volume. Similar to the EDSM with spatial replicates analysis, we considered all tows within the same subregion on the same day to be replicate surveys at a sample unit for the SKT data (Figure 1). This resulted in a dataset with 2031 sample units with an average of 1.71 replicate tows (range: 1–5) at each sample unit. The SKT tows can sometimes be spaced out relatively far in time even if they are conducted in the same subregion on the same day. Therefore, we considered an analysis that implemented a time restriction to only included tow data if the tow was conducted within a similar time span across temporal replicate surveys at a sampling location for EDSM. However, preliminary analyses yielded nearly identical results between the two approaches. Therefore, we did not implement a time restriction to filter the SKT data for our final analyses that we present herein.

We downloaded supplemental environmental data that may influence the distribution and detectability of Delta Smelt and Longfin Smelt in addition to the environmental data collected during trawl surveys. Estimates of X2 were calculated using Dayflow, which is a computer program that was developed to determine daily historical delta boundary hydrology (California Department of Water Resources. Accessed May 11, 2020, https://water.ca.gov/Programs/Environmental‐Services/Compliance‐Monitoring‐And‐Assessment/Dayflow‐Data). Time since sunrise is a covariate that may capture heterogeneity in detection probabilities across the day. It is similar to the recorded time of each tow, but it may be more biologically relevant since species often adjust behavior in response to daylight and the time of sunrise is variable across the year. We downloaded sunrise times for Rio Vista, CA using the R package *StreamMetabolism* (Sefick, 2016), which uses maptools based on the NOAA sunrise–sunset calculator. We used these sunrise data to calculate time since sunrise for all tows. Finally, how sample units are spatially arranged can have a large influence on the occurrence of fishes in the Bay‐Delta in addition to the environmental covariates normally collected and evaluated for these species (Peterson & Barajas, 2018). This can be related to environmental conditions not captured by the other covariates that are correlated with space, such as bottom depth, spatial proximity to other sample units, etc. Thus, we measured distance between the tow location and nearest shoreline to try to account for some of this unmodeled spatial environmental heterogeneity. For shoreline location, we downloaded the National Hydrography Dataset (NHD) High Resolution spatial data (U.S. Geological Survey. Accessed March 11, 2020, https://www.usgs.gov/core‐science‐systems/ngp/national‐hydrography/national‐hydrography‐dataset?qt‐science_support_page_related_con=0#qt‐science_support_page_related_con). The NHD represents the water drainage network in the USA with features such as rivers, streams, canals, lakes, ponds, coastline, dams, and stream gages. We downloaded these data for the State of California, clipped the shapefile to the area of interest, dissolved all polygons into a single polygon, and visually inspected the final shapefile to ensure there were no apparent issues. We then calculated the nearest distance between each tow location and the shoreline. Spatial analyses were completed in Esri ArcMap software (version 10.7.1).

### 2.4 Statistical analyses

We estimated the distribution of Delta Smelt and Longfin Smelt while accounting for incomplete capture using occupancy models (MacKenzie et al., 2002). Thus, our models estimated the probability that a sample unit is occupied by a species (*ψ_j_*_,_ *_k_*) and the probability that the species is detected, given that the sample unit is occupied by the species (*p_i_*_,_ *_j_*_,_ *_k_*), where *i*, *j*, and *k* denote tow, location, and tow set, respectively. Notably, occupancy models do not require that the same number of replicate tows occur in every sample unit. Furthermore, removal designs similar to the EDSM sampling protocol can be accommodated with this analytical approach, although the precision of the estimates can be less when compared to standard occupancy modeling sampling protocols (MacKenzie & Royle, 2005). Both of the probabilities described above can be related to explanatory variables using logit‐linear models (Duarte et al., 2019). Note that a central assumption of occupancy models is that the occupancy state across replicate surveys is static. Thus, explanatory variables cannot vary by tow for occupancy probability (*ψ*), but may vary by tow for detection probability (*p*). In cases where the explanatory variable was measured for each tow, we used the mean value (ignoring missing tow‐specific values) as a covariate for occupancy probability and the tow‐specific value as a covariate for detection probability.

We aimed to consider similar explanatory variables across species, datasets, and analyses, but this was not always possible (Table 1). Here, we describe the global model for the primary EDSM analysis first, followed by a description of the adjustments we made for the EDSM with spatial replicates analysis and then the SKT analysis. We evaluated if detection probability (*p*) was related to tow volume, secchi depth, tow number, time since sunrise, days since EDSM was initiated, dominant tide direction, and season. We note that days since EDSM was initiated was meant to capture potential learning (i.e., field crews may catch more fish as they gain experience in the field). It is also worth noting that season differed by species based on Merz et al. (2013) and Moyle et al. (2016), and this covariate was meant to represent potential differences in detection probability related to the dominant life stage that is available for capture. We modeled occupancy probability (*ψ*) as a function of day of year, the quadratic effect of day of year, mean temperature, X2, mean dissolved oxygen, mean secchi depth, mean distance to shoreline, mean specific conductivity, and the region within the Bay‐Delta (i.e., North, East, West, and Far West). The inclusion of day of year on occupancy probability was meant to represent the fluctuations in the number of fish that can be sampled by the sampling gear (i.e., the effective population that is being monitored) as fish progress through their life cycle. We did not consider tow duration, bottom depth, turbidity, and time of day as covariates because these variables were highly correlated (|*r*| > .75) with other explanatory variables of interest. Furthermore, we did not consider the average fish length per day as a covariate on detection probability because of the large number of missing values (Longfin Smelt = 70.2% missing; Delta Smelt = 64.7% missing) and the fact that this variable is closely tied to the season the surveys took place given the life history of these species. The EDSM with spatial replicates analyses were identical except we did not consider models that had both mean temperature and the quadratic effect of day of year on occupancy probability (*ψ*) because these variables were highly correlated in these data. We held the tow number to be consistent with the primary EDSM analyses because this covariate was meant to capture trends (either increasing or decreasing) in capture efficiency across replicate tows at a sampling location. The analyses of the SKT data differed slightly than the primary EDSM analyses. For detection probability (*p*), we no longer incorporated season since all tows occurred in the same season and we replaced day since EDSM was initiated with year since the SKT was initiated. Dissolved oxygen was not measured for SKT surveys, so we did not consider it in the SKT analyses. Furthermore, we never considered day of year and mean temperature in the same model for occupancy probability (*ψ*) because they were highly correlated in the SKT data. Preliminary analyses indicated the SKT Delta Smelt data were too sparse to incorporate mean temperature on occupancy probability (*ψ*), so we did not include this variable when analyzing these data. Similarly, preliminary analyses indicated the SKT Longfin Smelt data were too sparse to incorporate mean specific conductivity on occupancy probability (*ψ*), so we did not include this variable when analyzing these data.

**TABLE 1:** Mean, standard deviation (in parentheses), range, and percent missing data (if applicable) for continuous explanatory variables and frequency for categorical explanatory variables included in the occupancy models to estimate the probability that a sample unit is occupied (*ψ*), and the probability that the species is detected, given that the sample unit is occupied (*p*) for Delta Smelt (*Hypomesus transpacificus*) and Longfin Smelt (*Spirinchus thaleichthys*) using data collected by Enhanced Delta Smelt Monitoring Program, Enhanced Delta Smelt Monitoring Program (with spatial replicates), and Spring Kodiak Trawl

Variable	Parameter	Enhanced delta smelt monitoring program	Enhanced delta smelt monitoring program (with spatial replicates)	Spring Kodiak Trawl
Day of survey	*p*	513.06 d (194.97) 201–834	513.06 d (194.97) 201–834	na
Day of year	*ψ*	192.00 d (119.71) 2–365	191.70 d (120.48) 2–365	69.60 d (39.36) 7–140
Mean dissolved oxygen	*ψ*	8.89 mg/L (1.24) 4.60–14.22 0.34% missing	8.93 mg/L (1.20) 6.16–13.99 0.21% missing	na
Mean distance to shoreline	*ψ*	312.095 m (556.85) 4.10–4883.46	335.64 m (550.25) 8.32–4428.98	183.28 m (248.87) 0.30–1584.34
Mean secchi depth	*ψ*	0.89 m (0.53) 0.06–2.00	0.88 m (0.50) 0.06–2.00	0.65 m (0.42) 0.07–2.00 0.49% missing
Mean specific conductivity	*ψ*	4112.4 μs/cm^3^ (6873.12) 41–37,281 0.21% missing	4415.68 μs/cm^3^ (6997.59) 51.8–36,577 0.14% missing	2522.5 μs/cm^3^ (5414.37) 52–33,840 0.05% missing
Mean temperature	*ψ*	15.44°C (4.86) 6.45–26.78 0.21% missing	15.36°C (4.83) 6.93–26.45 0.14% missing	13.28°C (3.40) 6.25–23.70
Region	*ψ*	Far West: 300 North: 782 South: 459 West: 828	Far West: 203 North: 397 South: 332 West: 518	Far West: 259 North: 355 South: 793 West: 624
Season	*p*	Longfin Smelt: Juvenile: 1016 Subadult/adult: 1353 Delta Smelt: Juvenile: 489 Subadult: 854 Adult: 1026	Longfin Smelt: Juvenile: 600 Subadult/adult: 850 Delta Smelt: Juvenile: 296 Subadult: 624 Adult: 530	na
Secchi depth	*p*	0.91 m (0.53) 0.06–2.00 0.09% missing	0.91 m (0.53) 0.06–2.00 0.09% missing	0.62 m (0.41) <0.01–2.00 0.37% missing
Tide direction	*p*	Ebb: 589 Flood: 801 Slack: 979	Ebb: 372 Flood: 464 Slack: 614	Ebb: 1344 Flood: 593 Slack: 94
Time since sunrise	*p*	3.27 h (1.67) −0.13 to 9.82	3.27 h (1.67) −0.13 to 9.82	3.57 h (1.93) −0.15 to 9.88 0.03% missing
Tow number	*p*	3.04 (1.65) 1–9	3.04 (1.65) 1–9	1.63 (0.89) 1–5
Tow volume	*p*	3335.55 m^3^ (1168.39) 0.337–9491.25 0.20% missing	3335.55 m^3^ (1168.39) 0.337–9491.25 0.20% missing	5922.90 m^3^ (1332.59) 107.80–9978.50 6.94% missing
X2	*ψ*	75.24 km (9.01) 49.02–86.34	75.04 km (8.80) 49.02–86.34	67.60 km (10.35) 41.10–85.40
Year of survey	*p*	na	na	9.69 year (4.99) 1–18

Note

“na” indicates that the explanatory variable was not considered in the analysis.

We used a sequential‐by‐submodel strategy for our model selection procedure due to the large number of explanatory variables we wanted to consider. In particular, we fit every combination of explanatory variables on detection probability (*p*) while holding occupancy probability (*ψ*) constant (i.e., an intercept model). We then evaluated submodel importance using Akaike's Information Criterion corrected for small sample size (AIC_c_; Burnham & Anderson, 2002), where an important submodel was one that had a ∆AIC_c_ less than five and no uninformative parameters (see Arnold, 2010). We then carried important submodels forward to the next step and repeated the procedure for occupancy probability (*ψ*). Our final model set was restricted to the submodels that were deemed important at each step of this process. Importantly, we used this approach because it has been shown to recover a substantial portion of the total AIC_c_ model weight, recover the top‐ranked model, and reduce the number of models fit by nearly half when compared to fitting every combination of covariates for occupancy analyses (Morin et al., 2020). For all analyses, we standardized continuous covariates to have mean of zero and a standard deviation of one prior to fitting models, and we imputed the mean value (i.e., zero) for all missing values. The respective reference categories for region, season, and tide were Far West, Subadult/Adult (Longfin Smelt) or Subadult (Delta Smelt), and Slack. We described model parameters by their mean, standard error, and 95% confidence interval. We also calculated odds ratios for model coefficients (Hosmer & Lemeshow 2000) and considered a covariate to be strongly influential if the 95% confidence interval did not overlap zero. We also conducted a bootstrap goodness‐of‐fit test using the most complex model in the final model set for each analysis. Specifically, we used the estimates from the most complex model to simulate 10,000 datasets, fit the model to those simulated data, and compared the estimated variance inflation factor $\left(\hat{c}=\text{deviance}/df\right)$ when fitting the model to the real data with the variance inflation factors when fitting the model to the simulated data to evaluate the possibility of observing a deviance as large as what was estimated with the real data. We implemented these analyses using program MARK (version 9.0; White & Burnham, 1999) called from program *R* (R Development Core Team, 2016) using the package *RMark* (version 2.2.7; Laake, 2013).

## 3 RESULTS

Although the sequential‐by‐submodel strategy we used for our model selection procedure resulted in a large number submodel combinations that were evaluated, the number of models in the final model set was relatively low across datasets (Tables 2 and 3). We had competing models for most datasets (i.e., multiple models with ∆AIC_c_ < 2); however, close inspection of the model output verified that the estimated model intercepts and coefficients were nearly identical across all competing models for each analysis. Thus, we based our inferences on the model with the greatest AIC_c_ weight for each analysis (Tables 4 and 5). Notably, the bootstrap goodness‐of‐fit tests indicated decent fit across all datasets for Delta Smelt (EDSM: *p* = .875; EDSM with spatial replicates: *p* = .953; SKT: *p* = .462) and Longfin Smelt (EDSM: *p* = .971; EDSM with spatial replicates: *p* = .463; SKT: *p* = .394).

**TABLE 2:** Akaike's Information Criterion corrected for small sample size (AIC_c_), change in AIC_c_ (∆AIC_c_), model weights (*w_i_*), and number of parameters (*k*) for the probability that a sample unit is occupied (*ψ*), and the probability that the species is detected, given that the sample unit is occupied (*p*) for Delta Smelt (*Hypomesus transpacificus*) when using data collected by Enhanced Delta Smelt Monitoring Program, Enhanced Delta Smelt Monitoring Program (with spatial replicates), and Spring Kodiak Trawl

Dataset	Model	*k*	AIC_c_	∆AIC_c_	*w_i_*
Enhanced delta smelt monitoring program	**M1:** ψ (Day of year + Day of year^2^ + Mean temperature + Mean specific conductivity + Region) p (Tow volume + Secchi depth + Time since sunrise + Season)	14	1511.11	0.00	0.654
	**M2:** ψ (Day of year + Day of year^2^ + Mean temperature + Mean specific conductivity) p (Tow volume + Secchi depth + Time since sunrise + Season)	11	1512.38	1.27	0.346
Enhanced Delta Smelt Monitoring Program (with spatial replicates)	**M1:** ψ (Day of year + Mean temperature + X2 + Mean secchi depth + Mean distance to shoreline + Mean specific conductivity) p (Tow volume + Secchi depth + Tow number + Time since sunrise + Season)	14	1510.54	0.00	0.118
	**M2:** ψ (Day of year + Mean temperature + X2 + Mean secchi depth + Mean distance to shoreline + Mean specific conductivity) p (Tow volume + Secchi depth + Time since sunrise + Season)	13	1510.95	0.41	0.096
	**M3:** ψ (Day of year + Mean temperature + X2 + Mean secchi depth + Mean specific conductivity) p (Tow volume + Secchi depth + Tow number + Time since sunrise + Season)	13	1511.54	1.00	0.072
	**M4:** ψ (Mean temperature + X2 + Mean secchi depth + Mean distance to shoreline + Mean specific conductivity) p (Tow volume + Secchi depth + Tow number + Time since sunrise + Season)	13	1511.66	1.12	0.067
	**M5:** ψ (Day of year + Mean temperature + X2 + Mean secchi depth + Mean specific conductivity) p (Tow volume + Secchi depth + Time since sunrise + Season)	12	1511.93	1.39	0.059
	**M6:** ψ (Mean temperature + X2 + Mean secchi depth + Mean distance to shoreline + Mean specific conductivity) p (Tow volume + Secchi depth + Time since sunrise + Season)	12	1512.14	1.60	0.053
	**M7:** ψ (Mean temperature + X2 + Mean secchi depth + Mean specific conductivity) p (Tow volume + Secchi depth + Tow number + Time since sunrise + Season)	12	1512.50	1.95	0.044
	**M8:** ψ (Mean temperature + Mean secchi depth + Mean distance to shoreline + Mean specific conductivity) p (Tow volume + Secchi depth + Tow number + Time since sunrise + Season)	12	1512.59	2.05	0.042
	**M9:** ψ (Day of year + Mean temperature + X2 + Mean secchi depth + Mean distance to shoreline + Mean specific conductivity) p (Tow volume + Secchi depth + Tow number + Season)	13	1512.69	2.15	0.040
	**M10:** ψ (Mean temperature + X2 + Mean distance to shoreline + Mean specific conductivity + Region) p (Tow volume + Secchi depth + Time since sunrise + Season)	14	1512.70	2.16	0.040
	**M11:** ψ (Mean temperature + X2 + Mean distance to shoreline + Mean specific conductivity) p (Tow volume + Secchi depth + Tow number + Time since sunrise + Season)	12	1512.70	2.16	0.040
	**M12:** ψ (Mean temperature + X2 + Mean distance to shoreline + Mean specific conductivity) p (Tow volume + Secchi depth + Time since sunrise + Season)	11	1512.75	2.21	0.039
	**M13:** ψ (Mean temperature + X2 + Mean distance to shoreline + Mean specific conductivity + Region) p (Tow volume + Secchi depth + Tow number + Time since sunrise + Season)	15	1512.81	2.27	0.038
	**M14:** ψ (Mean temperature + X2 + Mean secchi depth + Mean specific conductivity) p (Tow volume + Secchi depth + Time since sunrise + Season)	11	1512.96	2.42	0.035
	**M15:** ψ (Mean temperature + Mean secchi depth + Mean distance to shoreline + Mean specific conductivity) p (Tow volume + Secchi depth + Time since sunrise + Season)	11	1513.13	2.59	0.032
	**M16:** ψ (Mean temperature + X2 + Mean specific conductivity) p (Tow volume + Secchi depth + Tow number + Time since sunrise + Season)	11	1513.48	2.94	0.027
	**M17:** ψ (Mean temperature + X2 + Mean specific conductivity) p (Tow volume + Secchi depth + Time since sunrise + Season)	10	1513.52	2.98	0.027
	**M18:** ψ (Day of year + Mean temperature + X2 + Mean secchi depth + Mean specific conductivity) p (Tow volume + Secchi depth + Tow number + Season)	12	1513.56	3.02	0.026
	**M19:** ψ (Mean temperature + X2 + Mean secchi depth + Mean distance to shoreline + Mean specific conductivity) p (Tow volume + Secchi depth + Tow number + Season)	12	1513.58	3.03	0.026
	**M20:** ψ (Mean temperature + X2 + Mean secchi depth + Mean specific conductivity) p (Tow volume + Secchi depth + Tow number + Season)	11	1514.28	3.74	0.018
	**M21:** ψ (Mean temperature + X2 + Mean distance to shoreline + Mean specific conductivity) p (Tow volume + Secchi depth + Tow number + Season)	11	1514.56	4.02	0.016
	**M22:** ψ (Mean temperature + Mean secchi depth + Mean distance to shoreline + Mean specific conductivity) p (Tow volume + Secchi depth + Tow number + Season)	11	1514.59	4.05	0.016
	**M23:** ψ (Mean temperature + X2 + Mean distance to shoreline + Mean specific conductivity + Region) p (Tow volume + Secchi depth + Tow number + Season)	14	1514.78	4.24	0.014
	**M24:** ψ (Mean temperature + X2 + Mean specific conductivity) p (Tow volume + Secchi depth + Tow number + Season)	10	1515.19	4.65	0.012
Spring Kodiak Trawl	**M1:** ψ (Day of year + Mean specific conductivity + Region) p (Survey year + Secchi depth + Tow number + Time since sunrise + Tide)	13	2865.06	0.00	1.000

**TABLE 3:** Akaike's Information Criterion corrected for small sample size (AIC_c_), change in AIC_c_ (∆AIC_c_), model weights (*w_i_*), and number of parameters (*k*) for the probability that a sample unit is occupied (*ψ*), and the probability that the species is detected, given that the sample unit is occupied (*p*) for Longfin Smelt (*Spirinchus thaleichthys*) when using data collected by Enhanced Delta Smelt Monitoring Program, Enhanced Delta Smelt Monitoring Program (with spatial replicates), and Spring Kodiak Trawl

Dataset	Model	*k*	AIC_c_	∆AIC_c_	*w_i_*
Enhanced Delta Smelt Monitoring Program	**M1:** ψ (Mean temperature + X2 + Mean secchi depth + Mean distance to shoreline + Region) p (Tow volume + Secchi depth + Season)	12	1350.55	0.00	0.456
	**M2:** ψ (X2 + Mean secchi depth + Mean distance to shoreline + Region) p (Tow volume + Secchi depth + Season)	11	1352.10	1.54	0.210
	**M3:** ψ (Mean temperature + X2 + Mean secchi depth + Mean specific conductivity + Region) p (Tow volume + Secchi depth + Season)	12	1352.37	1.82	0.184
	**M4:** ψ (Mean temperature + X2 + Mean secchi depth + Region) p (Tow volume + Secchi depth + Season)	11	1353.64	3.08	0.098
	**M5:** ψ (X2 + Mean secchi depth + Region) p (Tow volume + Secchi depth + Season)	10	1354.87	4.31	0.053
Enhanced Delta Smelt Monitoring Program (with spatial replicates)	**M1:** ψ (Mean temperature + X2 + Mean dissolved oxygen + Mean secchi depth + Region) p (Tow volume + Secchi depth + Season)	12	1376.41	0.00	0.358
	**M2:** ψ (Mean temperature + X2 + Mean secchi depth + Region) p (Tow volume + Secchi depth + Season)	11	1377.02	0.61	0.264
	**M3:** ψ (Mean temperature + Mean secchi depth + Mean specific conductivity + Region) p (Tow volume + Secchi depth + Season)	11	1377.13	0.73	0.249
	**M4:** ψ (X2 + Mean secchi depth + Region) p (Tow volume + Secchi depth + Season)	10	1378.45	2.04	0.129
Spring Kodiak Trawl	**M1:** ψ (Day of year + Day of year^2^ + X2 + Region) p (Secchi depth)	9	1355.22	0.00	0.725
	**M2:** ψ (X2 + Region) p (Secchi depth)	7	1357.16	1.94	0.275

**TABLE 4:** Mean, standard error (SE), and 95% confidence interval (CI) for the probability that a sample unit is occupied (*ψ*), and the probability that the species is detected, given that the sample unit is occupied (*p*) for Delta Smelt (*Hypomesus transpacificus*) when using data collected by Enhanced Delta Smelt Monitoring Program, Enhanced Delta Smelt Monitoring Program (with spatial replicates), and Spring Kodiak Trawl

Parameter	Enhanced delta smelt monitoring program				Enhanced delta smelt monitoring program (with spatial replicates)				Spring Kodiak Trawl			
	Mean	SE	Lower CI	Upper CI	Mean	SE	Lower CI	Upper CI	Mean	SE	Lower CI	Upper CI
*ψ*												
Intercept	−2.65	0.56	−3.75	−1.56	0.50	0.56	−0.60	1.60	−0.19	0.29	−0.77	0.38
Day of year	0.12	0.21	−0.29	0.53	−0.42	0.24	−0.89	0.06	−0.80	0.13	−1.05	−0.55
Day of year^2^	2.11	0.49	1.14	3.07	–	–	–	–	–	–	–	–
Mean temperature	2.28	0.41	1.47	3.09	0.93	0.22	0.50	1.36	na	na	na	na
X^2^	–	–	–	–	0.45	0.20	0.05	0.85	–	–	–	–
Mean secchi depth	–	–	–	–	0.95	0.51	−0.06	1.95	–	–	–	–
Mean distance to shoreline	–	–	–	–	−0.36	0.22	−0.79	0.07	–	–	–	–
Mean specific conductivity	−0.70	0.20	−1.09	−0.31	−0.62	0.20	−1.01	−0.24	−0.67	0.14	−0.95	−0.39
Region: North	0.13	0.54	−0.93	1.19	–	–	–	–	2.51	0.68	1.17	3.85
Region: South	−2.36	1.08	−4.47	−0.25	–	–	–	–	−0.31	0.38	−1.05	0.43
Region: West	−0.22	0.41	−1.02	0.58	–	–	–	–	1.48	0.32	0.85	2.11
*p*												
Intercept	−4.41	0.30	−5.00	−3.82	−4.78	0.29	−5.35	−4.20	−1.28	0.39	−2.03	−0.52
Tow volume	−0.17	0.10	−0.35	0.02	−0.19	0.09	−0.37	−0.02	–	–	–	–
Secchi depth	−3.19	0.26	−3.70	−2.68	−3.26	0.25	−3.76	−2.77	−1.18	0.10	−1.37	−0.98
Tow number	–	–	–	–	−0.15	0.10	−0.35	0.04	0.11	0.05	0.00	0.21
Time since sunrise	−0.27	0.11	−0.48	−0.06	−0.20	0.10	−0.40	−0.01	−0.25	0.06	−0.36	−0.14
Season: Juvenile	0.44	0.25	−0.05	0.92	0.24	0.22	−0.19	0.67	na	na	na	na
Season: Adult	−1.05	0.47	−1.96	−0.14	−1.26	0.32	−1.89	−0.63	na	na	na	na
Survey year	na	na	na	na	na	na	na	na	−0.76	0.06	−0.88	−0.65
Tide: Flood	–	–	–	–	–	–	–	–	0.74	0.39	−0.03	1.51
Tide: Ebb	–	–	–	–	–	–	–	–	0.15	0.39	−0.60	0.91

Note

“na” indicates that the explanatory variable was not considered in the analysis, and “–” indicates that the explanatory variable was included in the analysis but was not in the selected model.

**TABLE 5:** Mean, standard error (SE), and 95% confidence interval (CI) for the probability that a sample unit is occupied (*ψ*), and the probability that the species is detected, given that the sample unit is occupied (*p*) for Longfin Smelt (*Spirinchus thaleichthys*) when using data collected by Enhanced Delta Smelt Monitoring Program, Enhanced Delta Smelt Monitoring Program (with spatial replicates), and Spring Kodiak Trawl

Parameter	Enhanced delta smelt monitoring program				Enhanced delta smelt monitoring program (with spatial replicates)				Spring Kodiak Trawl			
	Mean	SE	Lower CI	Upper CI	Mean	SE	Lower CI	Upper CI	Mean	SE	Lower CI	Upper CI
*ψ*												
Intercept	−4.46	0.51	−5.46	−3.46	−2.46	0.55	−3.54	−1.37	1.38	0.80	−0.19	2.96
Mean temperature	−0.49	0.24	−0.96	−0.01	−0.69	0.28	−1.23	−0.14	–	–	–	–
Day of year	–	–	–	–	–	–	–	–	−0.07	0.19	−0.44	0.30
Day of year^2^	–	–	–	–	–	–	–	–	0.45	0.20	0.06	0.85
X^2^	0.50	0.13	0.25	0.75	0.38	0.15	0.09	0.67	1.04	0.28	0.50	1.58
Mean dissolved oxygen	–	–	–	–	−0.29	0.18	−0.65	0.07	na	na	na	na
Mean secchi depth	−3.59	0.50	−4.58	−2.61	−2.02	0.54	−3.07	−0.96	–	–	–	–
Mean distance to shoreline	0.20	0.09	0.03	0.37	–	–	–	–	–	–	–	–
Region: North	−2.29	0.49	−3.25	−1.32	−2.12	0.52	−3.14	−1.10	−3.73	0.90	−5.50	−1.96
Region: South	−1.55	0.80	−3.12	0.03	−1.72	0.81	−3.31	−0.12	−5.26	1.20	−7.62	−2.90
Region: West	−0.17	0.29	−0.73	0.40	−0.16	0.31	−0.77	0.45	−0.98	0.68	−2.31	0.34
*p*												
Intercept	−1.29	0.50	−2.27	−0.31	−3.34	0.46	−4.24	−2.45	−3.12	0.22	−3.55	−2.68
Tow volume	0.25	0.10	0.05	0.45	0.24	0.09	0.06	0.42	–	–	–	–
Secchi depth	−0.47	0.43	−1.31	0.36	−1.89	0.39	−2.66	−1.13	−2.85	0.27	−3.37	−2.32
Season: Juvenile	−1.64	0.43	−2.48	−0.80	−1.33	0.39	−2.10	−0.56	na	na	na	na

Note

“na” indicates that the explanatory variable was not considered in the analysis, and “–” indicates that the explanatory variable was included in the analysis but was not in the selected model.

**FIGURE 2:**
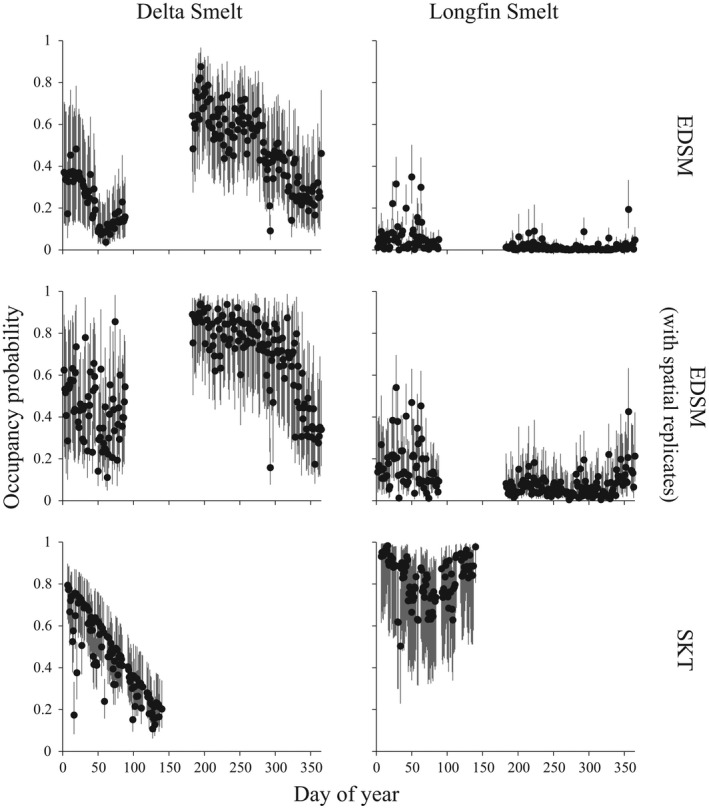
The estimated on average occupancy probability across the year for Delta Smelt (*Hypomesus transpacificus*) and Longfin Smelt (*Spirinchus thaleichthys*) when using data collected by Enhanced Delta Smelt Monitoring Program, Enhanced Delta Smelt Monitoring Program (with spatial replicates), and Spring Kodiak Trawl. Note that the average daily covariate information collected was used to generate daily predicted values and missing data points correspond to days not sampled by each monitoring program

The primary EDSM analysis estimated Delta Smelt occupancy probability was, on average, very low across regions (Table 4). Specifically, occupancy probability was, on average, 0.07 in Far West, with the odds of occupancy being 1.14 times higher in North, 10.59 times lower in South, and 1.25 times lower in West. Although, the relationship between occupancy and region was only strong for South, given all other coefficient estimates related to region had a confidence interval that overlapped zero. Occupancy probability had a positive relationship with day of year and the quadratic effect of day of year. The odds of occupancy were 9.78 times higher with every 4.86°C increase (1 SD) in mean temperature and 2.01 times lower for every 6873.12 μs/cm^3^ increase in mean specific conductivity. Collectively, this resulted in the on average occupancy probability being lowest late in the season when adults are the dominant life stage present and highest when juveniles are the dominant life stage present (Figure 2). The probability of detecting Delta Smelt given they were present was quite low (Table 4). Specifically, detection probability was, on average, 0.01. The odds of detection were 1.31 and 24.29 times lower for every 1.67 h past sunrise and 0.53 m increase in secchi depth, respectively. Furthermore, the odds of detection were 2.86 times lower in the season when adults are the dominant life stage present. Although detection probability was positively related to the juvenile season and negatively related to tow volume, these effects were weak with the confidence interval for the coefficient estimates overlapping zero.

The EDSM with spatial replicates analysis estimated Delta Smelt occupancy probability was, on average, relatively high (0.62) and, unlike the primary EDSM analysis, did not vary across regions (Table 4). Occupancy probability still had a strong, positive relationship with mean temperature and a strong, negative relationship with mean specific conductivity; however, now occupancy probability was also found to have a strong, positive relationship with X2. Specifically, the odds of occupancy were 2.53 times higher with every 4.83°C increase in mean temperature, 1.57 times higher with every 8.80 km increase in X2, and 1.86 times lower with every 6997.59 μs/cm^3^ increase in mean specific conductivity. Although occupancy now increased with mean secchi depth and decreased with day of year and mean distance to shoreline, these relationships were not strong because the confidence intervals for the coefficient estimates overlapped zero. Although higher for this analysis, the on average occupancy probability decreased late in the year, which was similar to the primary EDSM analysis (Figure 2). Still, contrary to the primary EDSM analysis, no discernable pattern in the on average occupancy probability was found during the season when adults are the dominant life stage present. Overall, patterns in detection probability were similar to before with a few exceptions (Table 4). Detection probability was still, on average, 0.01. Similarly, the odds of detection were 1.22 times lower for every 1.67 h past sunrise, 26.05 times lower for every 0.53 m increase in secchi depth, and 3.53 times lower in the season dominated by the adult life stage. Detection probability was now strongly related to tow volume, with the odds of detection being 1.21 times lower with every 1168.39 m^3^ increase in tow volume. Although detection probability was now negatively related to tow number, the effect was weak with the confidence interval for the coefficient estimate overlapping zero. The effect of the juvenile season continued to be weak.

The SKT analysis also estimated Delta Smelt occupancy probability was, on average, relatively high compared to the primary EDSM analysis (Table 4). Specifically, occupancy probability was, on average, 0.45 in Far West, with the odds of occupancy being 12.30 times higher in North, 1.36 times lower in South, and 4.39 times higher in West. However, the relationship between occupancy and region was not strong for South, given the coefficient estimate overlapped zero. The effect of mean specific conductivity on occupancy was similar to the other analyses but occupancy now had a strong, negative relationship with day of year. Specially, the odds of occupancy were 1.95 times lower with every 5414.37 μs/cm^3^ increase in mean specific conductivity and 2.23 times lower with every 39.36 days passed. Although higher for this analysis when compared to the primary EDSM analysis, the on average occupancy probability was still lowest late in the season when adults are the dominant life stage present (Figure 2). Here, detection probability was relatively high, with the on average detection probability estimated at 0.22 (Table 4). Detection probability still had a strong, negative relationship with secchi depth and time since sunrise. The odds of detection were 1.28 and 3.25 times lower for every 1.93 h past sunrise and 0.41 m increase in secchi depth, respectively. Detection probability now increased with tow number with the odds of detection being 1.12 times higher with every 0.89 tows passed. Detection probability also had a strong, negative relationship with survey year (a covariate not considered in EDSM analyses) with the odds of detection being 2.14 times lower after every 4.99 years. Although tide direction was in the selected model, the effect of this covariate was not strong given the confidence interval for both coefficient estimates overlapped zero.

The primary EDSM analysis estimated the on average occupancy probability for Longfin Smelt was lower than Delta Smelt (Table 5). Specifically, occupancy probability was 0.01, on average, in Far West. The odds of occupancy were 9.87 times lower in North, 4.71 times lower in South, and 1.19 times lower in West. It seems regional variation in occupancy was governed by the difference between Far West and North, given the confidence intervals for the other coefficient estimates overlapped zero. Occupancy was positively related to X2 and mean distance to shoreline, but negatively related to mean temperature and mean secchi depth. In particular, the odds of occupancy were 1.63 times lower with every 4.86°C increase in mean temperature, 1.65 times higher with every 9.01 km increase in X2, 36.23 times lower with every 0.53 m increase in mean secchi depth, and 1.22 times higher with every 556.85 m increase in mean distance to shoreline. Collectively, this led to the on average occupancy probability being low across most of the year with some higher estimates in the season when adults and subadults are the dominant life stage present (Figure 2). The probability of detecting Longfin Smelt given they were present was, on average, 0.22. The odds of detection were 1.28 times higher with every 1168.39 m^3^ increase in tow volume and 5.16 times lower in the season when juveniles are the dominant life stage available for capture. The effect of secchi depth was not strong since the confidence interval overlapped zero.

The EDSM with spatial replicates analysis estimated Longfin Smelt occupancy probability was relatively high when compared to the primary EDSM analysis (Table 5). Occupancy probability was 0.08, on average, in Far West. The odds of occupancy were 8.33 times lower in North, 5.58 times lower in South, and 1.17 times lower in West. Again, the confidence interval for the coefficient estimate representing West overlapped zero, indicating occupancy probability in Far West and West were similar. Occupancy still had a strong, negative relationship with mean temperature and mean secchi depth and a strong, positive relationship with X2. The odds of occupancy were 1.99 times lower with every 4.83°C increase in mean temperature, 1.46 times higher with every 8.80 km increase in X2, and 7.54 times lower with every 0.50 m increase in mean secchi depth. Although mean dissolved oxygen was now included in the selected model for occupancy probability, the relationship was not strong because the confidence interval for the coefficient estimate overlapped zero. Mean distance to shoreline was no longer in the selected model. Although relatively high compared to the primary EDSM analysis, similar patterns in the on average occupancy probability were found across the year (Figure 2). Detection probability was much lower for this analysis, with the on average detection probability estimated at 0.03 (Table 5). Still the relationships between detection probability and the covariates considered were similar to the primary Longfin Smelt EDSM analysis. The odds of detection were 1.27 times higher with every 1168.39 m^3^ increase in tow volume and 3.78 times lower in the season when juveniles are the dominant life stage available for capture. The effect of secchi depth on detection probability was now strong, with the odds of detection being 6.62 times lower with every 0.53 m increase in secchi depth.

The SKT analysis estimated Longfin Smelt occupancy probability was relatively high compared to both of the other analyses for this species (Table 5). Occupancy probability was 0.80, on average, in Far West. The odds of occupancy were 41.68 times lower in North, 192.48 times lower in South, and 2.66 times lower in West. Again, the confidence interval for the coefficient estimate representing West overlapped zero, indicating occupancy probability in Far West and West regions were similar. This analysis indicated occupancy probability was only related to region, day of year, and X2. The odds of occupancy were 2.83 times higher with every 10.35 km increase in X2. The on average occupancy probability pattern across the season was quite different than the other two Longfin Smelt analyses, with the nadir occurring mid‐season (Figure 2). The on average detection probability was estimated at 0.04, which is similar to the Longfin Smelt EDSM with spatial replicates analysis (Table 5). For this analysis, detection probability was only related with secchi depth. The odds of detection were 17.29 times lower with every 0.41 m increase in secchi depth.

## 4 DISCUSSION

Occupancy models are increasingly used to adjust monitoring data for imperfect detection to more accurately track how and why species redistribute across dynamic landscapes; however, many long‐term monitoring programs lack the temporal replicate surveys at a sample unit needed to fit these models using standard approaches. We analyzed Delta Smelt and Longfin Smelt monitoring data that use temporal and spatial replicate surveys to demonstrate some of the tradeoffs associated with implementing a space‐for‐time substitution with occupancy models. In doing so, we also evaluated the influence of environmental and sampling conditions that are related to the distribution and detectability of these species of concern.

The probability a sample unit was occupied was much higher when using spatial replicates for both species and both datasets. This finding was expected and is likely related to the change in the spatial scale of our inferences. Subregions represented a sample unit when using spatial replicates, which is considerably larger than the area around a EDSM sampling location. As the spatial extent of a sample unit increases the likelihood a species is present in the sample unit concomitantly increases (i.e., the species area relationship; Connor & McCoy 1979). Admittedly, it is not clear to us how occupancy dynamics at the scale of an entire subregion will help inform real‐time management decision making for these species in the Bay‐Delta. We treated subregions as sample units for the spatial replicate analyses because these delineations are used by managers in the Bay‐Delta for planning purposes, this approach provided the number of replicate surveys needed based on the fixed locations of the SKT survey stations, and this scale of inference is being used for other species, in particular Chinook Salmon (*Oncorhynchus tshawytscha*), in the Bay‐Delta (Mahardja et al., 2021). If this scale of inference is deemed suitable for informing management decision making for Delta Smelt and Longfin Smelt, it is worth noting that how stations are selected for surveys matters. Indeed, simulation studies show that selecting stations for surveys without replacement may induce dependence in the data that leads to biased estimates of occupancy and misleading temporal trends (Kendall & White, 2009; MacKenzie et al., 2017). Importantly, SKT data were sampled without replacement when treating stations as spatial replicate surveys, and we could not retroactively fix this potential issue in the survey data. This may partly explain the differences in the estimates when comparing results from the EDSM and SKT analyses. Thus, it would be worthwhile to modify the existing SKT sampling design to select survey stations within a subregion with replacement if this spatial resolution for occupancy dynamics will be considered further with these data.

As the spatial extent of our sample units increased, our ability to detect environmental effects on the distribution of species became complicated. In particular, the relationship between occupancy and explanatory variables differed by analysis, with some variables no longer having support and others having the opposite effect. Although both monitoring programs use the same sampling gear, it seems plausible that some of these patterns are related to the differences in spatial and temporal coverage between the two monitoring programs. That is, the different surveys may be sampling different segments of the populations. However, differences between the results of EDSM analyses that used the same exact data were also apparent. Thus, we suspect these patterns are also largely related to the tradeoffs associated with spatial grain and extent. As noted earlier, a fundamental assumption of occupancy models is that the occupancy state does not change across replicate surveys. This meant that tow‐specific measurements of environmental conditions had to be combined across replicate tows to include as covariates on occupancy probability. However, as the spatial extent of sample units increased the environmental covariates we considered likely displayed more heterogeneity within a sample unit (Wiens, 1989). Although standard practice, the combined value we used to capture environmental conditions (i.e., the mean) did not capture this heterogeneity and by extension likely were not representative of habitat conditions within a sample unit at the scale of a subregion. Thus, careful consideration needs to be given concerning the heterogeneity of environmental conditions within sample units when determining the appropriate spatial extent of sample units and how to summarize covariate information.

Inferences regarding detection probabilities also differed considerably between spatial and temporal replicate survey designs. When there are temporal replicate surveys within a sample unit, detection probabilities are interpreted as the probability of detecting a species given it occurs in the sample unit. In contrast, when using spatial replicates within a sample unit the detection probabilities estimate the probability of detecting a species at a tow location given they occur within the subregion at that tow location (i.e., availability for capture). The probability of detecting a species in a sample unit depends on three things: the unit is occupied, the probability of capturing at least one individual, and the number of individuals available for capture (Bayley & Peterson, 2001; Royle & Nichols, 2003). Thus, the observed decrease in Delta Smelt detection probabilities through time for SKT could be due to at least three potential mechanisms working individually or in concert. First, the decrease in detection probability could be due to a contraction in the distribution of a species within subregions leading to fewer SKT stations where Delta Smelt are present. Second, the decrease in detection probability could be due to an environmental change over time, such as water becoming clearer, that concomitantly reduces fish capture efficiency with nets. Finally, the decrease in detection probability could be due to overall decreases in Delta Smelt abundance and thus, fewer fish available for capture. Although we are unable to determine the mechanism(s) responsible, this finding suggests that attempts to adjust historical SKT data with contemporary detection probability models may not be a valid approach.

This leads to the question: are we overly relying on occupancy models for the sake of using them when many long‐term monitoring programs were established without considering this analytical approach? The answer to this question depends on the objectives of the monitoring program, the ecology of the species, the characteristics of its existing sampling design, and the willingness of managers to modify the existing sampling design if needed. The SKT and EDSM were both initiated with the aim of monitoring the distribution and relative abundance of Delta Smelt to inform policy and management decision making in the Bay‐Delta. It is clear that monitoring data collected in the Bay‐Delta are complicated by the observation process and that capture efficiency can vary considerably (Goertler et al., 2020; Latour, 2016; Mahardja et al., 2021, 2017; Mitchell et al., 2017, 2019; Newman, 2008; Peterson & Barajas, 2018; Polansky et al., 2019; Thomson et al., 2010; this study). Although trends in occupancy and relative abundance of fishes in the raw catch data for a few species and monitoring programs (with different sampling extents and resolutions) in the Bay‐Delta appear unbiased when compared to estimates from multistate occupancy models (Peterson & Barajas, 2018), there are at least two primary reasons why not separating the ecological and observation process is problematic with respect to the goals of SKT, EDSM, and management in the Bay‐Delta. First, the point estimates are systematically lower when not accounting for incomplete capture. This means that estimates of distribution and relative abundance of fishes depict a scenario that is much grimmer than reality when not accounting for incomplete capture. Although there seems to be a general sense that erring on the side of being conservative (i.e., estimating the species is worse off than reality) is less problematic when managing at‐risk species, management in the Bay‐Delta is complex and needs to consider the objectives of multiple stakeholder groups. Being less transparent in the status of species or using less reliable information to inform policy and management decisions can understandably lead to mistrust in the decision‐making process, which can have negative ramifications for species conservation over the long term (Conroy & Peterson, 2013; Gregory et al., 2012). Second, the agreement between the trends in raw catch data and occupancy estimates while accounting for incomplete capture will diminish if detection probabilities become more variable as system‐wide environmental changes in the Bay‐Delta continue to progress. This is especially the case if the environmental factor that changes influences the ecological and observation process in opposite directions. There is no shortcut to evaluating the effect of detection probabilities on inferences. One has to estimate detection probability to know how big of a problem it is. Thus, it is our view that analytical methods that account for incomplete capture are necessary to reliably track the distribution and relative abundance of species to inform policy and management decision making.

Occupancy models are a robust approach to correct long‐term monitoring data for incomplete capture to reliably estimate the distribution of species, but, as we noted earlier, their use does not preclude the need for a robust sampling design. The exact number of replicate surveys needed depends on the system that is being monitored (i.e., *ψ* and *p*; variation in these parameters; and the number of sample units and years of monitoring; MacKenzie et al., 2017). It should be noted that collecting the additional data needed to fit occupancy models to evaluate the effect of detection probabilities on inferences can be incorporated into long‐term monitoring programs, such as the SKT, while maintaining data continuity by modifying existing sampling designs to include temporal replicate surveys at a survey station. Unfortunately, there are often barriers to modifying or replacing established monitoring programs even after it is clear that they implement a less than optimal sampling design to reliably track the system states of interest (reviewed in Thompson et al., 1998). The initiation of EDSM represents a potential unforeseen opportunity given the EDSM Kodiak trawl uses the same gear as SKT and EDSM incorporates temporal replicate surveys at a sampling location. In particular, integrated analyses may allow SKT data to be corrected for incomplete capture without modifying the SKT sampling protocol or extent of the sample unit if it can be assumed that detection probabilities do not differ substantially between the monitoring programs (Grabowski et al., 2009). Again, we caution against using this approach for historical SKT data without a proper evaluation of its validity given our results indicate system‐wide changes may have occurred such that detection probabilities for historical SKT data are not comparable to detection probabilities for EDSM data. Additionally, occupancy analyses of these historical data would benefit from more integrated analyses that leverage data from the multiple monitoring programs while treating the different sampling gears as different detection methods directly in the model (Nichols et al., 2008). To our knowledge, Mahardja et al. (2021) were the first to try this in the Bay‐Delta when analyzing Chinook Salmon detection/non‐detection data at the spatial scale of a subregion. Our results suggest this approach requires more careful consideration to identify a smaller spatial scale when the objective is to relate occupancy probability to environmental covariates. Although the appropriate spatial scale is admittedly hard to define, we propose such an approach would likely require some consideration of the following: the spatial and temporal overlap in the monitoring programs to effectively borrow information across datasets; the movement ecology of the focal species to meet model assumptions of closure and independence (or perhaps use estimators that account for dependence in the data); the heterogeneity in environmental conditions to accurately represent habitat within sample units; and the scale at which information is used to inform management decisions to provide information that is directly relevant to managers and policy makers.

A primary objective of many long‐term monitoring programs, including the trawl surveys in the Bay‐Delta, is to track the distribution and relative abundance of species in relation to changing environmental conditions. N‐mixture models have increasingly been used to analyze count data such as these to estimate spatial and temporal patterns in abundance while accounting for imperfect detection; however, these models are extremely sensitive to assumption violations and unmodeled heterogeneity in the monitoring data (Barker et al., 2017; Duarte et al., 2018; Link et al., 2018). This is the primary reason we opted to fit occupancy models in this study. Although occupancy probabilities can be positively correlated with abundance (Clare et al., 2015; Collier et al., 2013; Linden et al., 2017; Mathewson et al., 2012), this relationship is not always supported (Ellis et al., 2013; Stauffer et al., 2021). It is worth highlighting the potential utility of multistate occupancy models to correct monitoring data for imperfect detection while estimating more than two occupancy states (Nichols et al., 2007). Specifically, occupancy states can be defined in terms of relative abundance: the probability that a sample unit is occupied and the probability that a sample unit is occupied by a large number of individuals (relatively abundant) given that the sample unit is occupied. This approach has proven useful for other systems and species (MacKenzie et al., 2009; Martin et al., 2010; Royle, 2004; Royle & Link, 2005; Whitlock et al., 2020) and for Bay‐Delta fishes specifically (Peterson & Barajas, 2018). Still, using multistate occupancy models to estimate occupancy and relative abundance is relatively underutilized in the literature, which is likely related to the lack of an in‐depth simulation‐based study to evaluate how this estimator performs under various sampling and environmental conditions. We encourage work focused on evaluating how robust relative abundance estimates from multistate occupancy models are to provide some level of credence (or lack thereof) to this approach to estimate the distribution and relative abundance of species from replicate count data.

Information concerning how and why species redistribute across dynamics landscapes is invaluable to help guide policy and management decisions. Thus, it comes as no surprise that managers have invested considerable resources to support long‐term monitoring programs. Our results demonstrate how analyzing real‐world monitoring data using occupancy models and spatial replicates within a sample unit can influence inferences regarding the distribution and detection probability of species. Overall, we found using spatial replicates within a sample unit was not a substitution at all, but instead changed the scale of our inferences enough that it complicated our ability to relate environmental conditions to the distribution of pelagic fishes in the Bay‐Delta. Our work highlights the importance of considering how the unique characteristics of monitoring programs, particularly the spatial extent of sample units, influences our inferences regarding the occurrence and detectability of species. Notably, even in the best of circumstances detecting a declining trend can take multiple years of monitoring, resulting in unnecessarily delayed and reactive management actions. This delay can potentially lead to system states that require a level of investment in resources that is beyond what is available to reverse species declines (reviewed in Grant et al., 2013). Thus, it would be worthwhile to translate results from analyses of monitoring data such as this into predictive models within a decision analytic framework to facilitate proactive management strategies and learning via adaptive management.

## CONFLICT OF INTEREST

None declared.

## AUTHOR CONTRIBUTION

**Adam Duarte:** Conceptualization (equal); Formal analysis (lead); Funding acquisition (equal); Writing‐original draft (lead); Writing‐review & editing (equal). **James T. Peterson:** Conceptualization (equal); Funding acquisition (equal); Writing‐review & editing (equal).

## Data Availability

All data used in this article are publicly available. The EDSM data can be accessed through the EDI Data Portal at https://portal.edirepository.org/nis/mapbrowse?packageid=edi.415.1 (Accessed August 27, 2019). The SKT data can be accessed through California Department of Fish and Wildlife at http://www.dfg.ca.gov/delta/projects.asp?ProjectID=SKT (Accessed April 15, 2020). Tide height data from the National Oceanic and Atmospheric Administration (NOAA) station at Port Chicago, CA can be accessed at https://tidesandcurrents.noaa.gov/stationhome.html?id=9415144 (Accessed May 11, 2020). Estimates of X2 can be accessed at https://water.ca.gov/Programs/Environmental‐Services/Compliance‐Monitoring‐And‐Assessment/Dayflow‐Data (Accessed May 11, 2020). National Hydrography Dataset (NHD) High Resolution spatial data can be accessed at https://www.usgs.gov/core‐science‐systems/ngp/national‐hydrography/national‐hydrography‐dataset?qt‐science_support_page_related_con=0#qt‐science_support_page_related_con (Accessed March 11, 2020).
